# Books and Authors

**Published:** 1910-02

**Authors:** 


**Progressive Medicine, Vol. IV, December, 1909.** A Quarterly Digest of Advances, Discoveries and Improvements in the Medical and Surgical Sciences. Edited by **Hobart Amory Hare**, M.D., Professor of Therapeutics and Materia Medica in the Jefferson Medical College of Philadelphia. Octavo, 334 pages, with 35 engravings and a colored plate. Per annum, in four paper-bound volumes, containing over 1,200 pages, $6.00, net; in cloth, $9.00, net. Lea & Febiger, Publishers, Philadelphia and New York.

The December number of this valuable serial contains articles of importance both to the general practitioner and specialist. They are, first, diseases of the digestive tract and allied organs, the liver and pancreas, by David L. Edsall; diseases of the kidneys, by John Rose Bradford; surgery of the extremities, tumors, surgery of joints, shock, anesthesia, and infections, by Joseph C. Bloodgood; genitourinary diseases, by William T. Belfield; and a practical therapeutic referendum, by H. R. M. Landis. Of special interest is Bloodgood's discussion of fractures and in particular his comments on Lane's open treatment method. He speaks with disfavor of the extension treatment of fracture of the neck of the femur,—an opinion with which most surgeons of experience will agree.

If one would keep up with the advances taking place so rapidly in medicine and surgery nowadays, it is pretty nearly a necessity to possess himself of "Progressive Medicine," which presents in quarterly installments, the newest and best, garnered from the choicest literature of the world's storehouses.

---

**Modern Clinical Medicine.** Diseases of Children. Edited by **Abraham Jacobi,** M.D., Consulting Physician to Mt. Sinai, Bellevue, Babies' and other hospitals, New York. Translated from "Die Deutsche Klinik" under the general editorial charge of **Julius L. Salinger,** M.D. Octavo, pp. 843. Illustrated. New York and London: D. Appleton & Co. 1910. (Cloth, $6.00.)

The list of contributors to this work comprises well-known men from Germany and Austria, while most of them are masterful exponents of the science upon which they discourse in this volume. When to all this is added the fact that the editor, Dr. Jacobi, is himself one of the most famous of American physicians, particularly skilled, too, in the special field of pediatrics, we have ample reason to justify the belief that this is one of the best of the many textbooks lately issued relating to diseases of children.

Keller deals with the newborn in the first days of life, a topic we particularly like, because authors generally do not make this classification and deal only tentatively with diseases at this period of life. Keller endorses Credé's method of prophylaxis against ophthalmia neonatorum; not only so but gives Credé's own directions as to the method of using the silver nitrate. Obstetricians everywhere should read this section and ponder it well. Keller says Credé's method is the only sure way to prevent the disease,—that there is no substitute.

Czerny discourses upon the feeding of children in a most instructive fashion; indeed, in a way that indicates full knowledge of the subject. The importance of feeding a child properly can scarcely be overestimated, since most diseases, save the acute exanthematous infections, may be prevented by a proper adaptation of food. It requires rare skill and judgment to regulate the feeding problem to the adequate nourishment of the infant without deranging the digestive tract. The acute digestive disturbance of infancy furnish some of the most difficult problems to be met in the sick rooms of children. It requires rare tact and judgment to deal with them, and it is one of the valuable features of this book that these conditions are so skilfully handled. The chronic diseases of the digestive tract, too, are ably treated. These two groups of diseases are well worth the study of every physician whose province it is to treat sick children.

Another important topic is hereditary syphilis, one which Finkelstein presents with rare acumen. It is a disease of a far reaching nature and oftentimes difficult to detect, especially where there is mixed infection. What Finkelstein says will prove of great aid in the diagnosis, particularly in the syphilis of nurselings.

The book is full of choice information and may be studied with benefit both by specialists and general practitioners. A word of praise should be given the translator who has reduced the text to smooth, delightful English, which is a genuine pleasure to read. A few illustrations, several of which are temperature charts, add to the usefulness of the book.

**Dose Book and Prescription Writing.** By **E. Q. Thornton,** M.D., Assistant Professor of Materia Medica, Jefferson Medical College, Philadelphia. Fourth edition revised. 12 mo of 410 pages, illustrated. Philadelphia and London: W. B. Saunders Company, 1909. (Flexible leather, $2.00 net.)

This book contains a vast amount of information that is useful to every physician. Indeed, it is a book that may be kept on the office desk with great propriety. The manual of prescription writing is one of the best we have seen; as a mere dose book, too, it is unexceled. It is divided into four parts besides an introduction and an appendix. The first part deals with weights and measures; the second, with prescription writing; the third, with official preparations and methods of prescribing; and the fourth, with dosage. In the appendix common poisons and their antidotes are presented; synonyms of common drugs and preparations are given, and a mass of other information of value is set forth. This revision makes the book about as complete as possible.

---

**A Manual of Normal Histology and Organography.** By **Charles Hill,** Ph.D., M.D., Formerly Assistant Professor of Histology and Embryology, Northwestern University Medical School, Chicago. Second revised edition. 12mo of 468 pages, with 312 illustrations. Philadelphia and London: W. B. Saunders Company, 1909. (Flexible leather, $2.00 net.)

Intended for elementary study the author has presented the fundamental facts pertaining to normal histology in a clear as well as concise manner. Moreover, he has made what is usually considered rather a dull subject, so attractive that it becomes a pleasure to pursue it. Hill says neglect of proper care of the teeth is a common failing,—a truism that will bear frequent repetition. He has dwelt with considerable force and detail on this topic. This revision has afforded the author an opportunity to rewrite some of the chapters, and to incorporate the more recent contributions to histology in the text of others. The illustrations are of the better order, serving to develop the text, and taken together both in text and figures, it constitutes one of the foremost manuals on histology in print.

